# Early post-transplant calciphylaxis in a renal allograft recipient: diagnostic and therapeutic challenges

**DOI:** 10.1590/2175-8239-JBN-2026-0187en

**Published:** 2026-11-06

**Authors:** Johanna Viana, Maria Inês Bessa Vasconcelos, Manuela Bustorff

**Affiliations:** 1 Unidade Local de Saúde de Braga, Departamento de Nefrologia, Braga, Portugal.; 2 Unidade Local de Saúde São João, Departamento de Nefrologia, Porto, Portugal.

**Figure 1:**
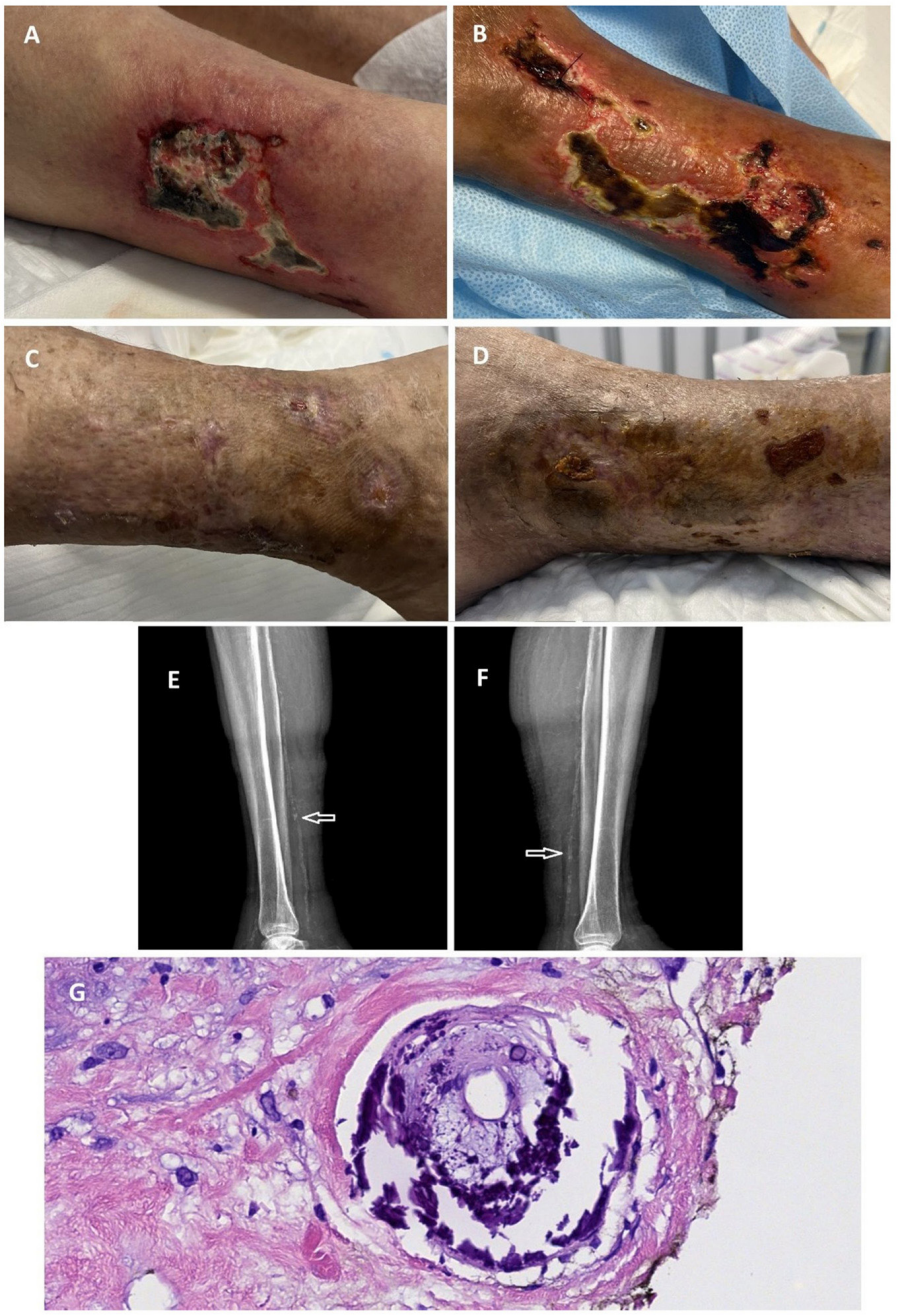
Necrotic ulcers on the right **(A)** and left **(B)** lower legs at presentation, showing central black eschars with surrounding erythematous borders, consistent with ischemic necrosis in the context of calciphylaxis. At three-month follow-up, near-complete resolution of the lesions on the right **(C)** and left **(D)** lower legs was observed, with residual scarring and no clinical evidence of active infection or recurrence. Plain radiographs of the right **(E)** and left **(F)** lower legs show peripheral vascular calcifications (arrows) distributed linearly along the arterial walls. Skin biopsy **(G)** with hematoxylin and eosin staining at 40× digital magnification exhibits a small subcutaneous vessel with extensive calcification of the vascular wall, intimal hyperplasia, and luminal occlusion by thrombotic material, findings characteristic of calcific uremic arteriolopathy.

A 59-year-old kidney transplant recipient with moderate graft dysfunction, on prednisolone, tacrolimus, and mycophenolic acid, presented 9 months post-transplant with a two-week history of extremely painful bilateral leg ulcers. His medical history included hemodialysis for 4 years and warfarin use until transplant. He denied trauma or diabetes. Parathyroid hormone was 130 pg/mL, with normal calcium and phosphate levels; the patient was not taking vitamin D or calcium-based phosphate binders. The immunological work-up was negative. Vascular studies excluded significant peripheral arterial disease but demonstrated diffuse calcification; a skin biopsy confirmed calciphylaxis—a rare yet potentially fatal post-transplant condition^1^. Multidisciplinary treatment, including intravenous sodium thiosulfate, infection management, debridement, and partial-thickness skin grafting, achieved near-complete healing within three months.

## Data Availability

No datasets were generated or analyzed during the current study. All relevant information is included in the manuscript.
