# Supplementary material for: Early post-transplant calciphylaxis in a renal allograft recipient: diagnostic and therapeutic challenges
Source: J Bras Nefrol. 2026 Nov 6;49(1):e20260187. doi: 10.1590/2175-8239-JBN-2026-0187en (PMC13743144; doi:10.1590/2175-8239-JBN-2026-0187en)
Supplement: Translation

# Translation: Calcifilaxia precoce pós-transplante em receptor de aloenxerto renal: desafios diagnósticos e terapêuticos

**DOI:** 10.1590/2175-8239-JBN-2026-0187pt

**Authors:** Johanna Viana, Maria Inês Bessa Vasconcelos, Manuela Bustorff

**Affiliations:** 1 Unidade Local de Saúde de Braga, Departamento de Nefrologia, Braga, Portugal.; 2 Unidade Local de Saúde São João, Departamento de Nefrologia, Porto, Portugal.

**Figura 1:** Úlceras necróticas na perna direita **(A)** e esquerda **(B)** no momento da apresentação, evidenciando escaras negras centrais, com bordas eritematosas circundantes, compatíveis com necrose isquêmica no contexto de calcifilaxia. Após três meses de tratamento, observa-se a resolução quase completa das lesões nas pernas direita **(C)** e esquerda **(D)**, com cicatrizes residuais e sem evidência clínica de infecção ativa ou de recorrência. As radiografias da perna direita **(E)** e esquerda **(F)** demonstram calcificações vasculares periféricas (setas), dispostas linearmente ao longo das paredes arteriais. A biópsia cutânea **(G)**, com coloração de hematoxilina-eosina e ampliação digital de 40×, revela um pequeno vaso subcutâneo com calcificação extensa da parede vascular, hiperplasia da íntima e oclusão luminal por material trombótico, achados característicos de arteriolopatia urêmica calcificante.

Um receptor de transplante renal de 59 anos com disfunção moderada do enxerto e em uso de prednisolona, tacrolimus e ácido micofenólico apresentou, 9 meses após o transplante, úlceras bilaterais extremamente dolorosas nas pernas, com 2 semanas de evolução. Apresentava antecedentes de hemodiálise durante 4 anos e uso de varfarina até o transplante. Negava trauma ou diabetes. O nível de paratormônio era de 130 pg/mL, com níveis normais de cálcio e fósforo; não havia uso de vitamina D nem quelantes de fósforo à base de cálcio. A avaliação imunológica foi negativa. O estudo vascular excluiu doença arterial periférica significativa, mas demonstrou calcificação vascular difusa. A biópsia cutânea confirmou o diagnóstico de calcifilaxia – uma entidade rara, mas potencialmente fatal no pós-transplante.^1^ Foi submetido a tratamento multidisciplinar, incluindo tiossulfato de sódio intravenoso, tratamento da infecção, desbridamento e enxerto cutâneo de espessura parcial, com cicatrização quase completa em três meses.

## Data Availability

Não foram gerados nem analisados quaisquer conjuntos de dados durante o presente estudo. Todas as informações relevantes estão incluídas no manuscrito.
